# Supplementary material for: Complementary Sets of Autoantibodies Induced by SARS-CoV-2, Adenovirus and Bacterial Antigens Cross-React with Human Blood Protein Antigens in COVID-19 Coagulopathies
Source: Int J Mol Sci. 2022 Sep 29;23(19):11500. doi: 10.3390/ijms231911500 (PMC9569991; doi:10.3390/ijms231911500)
Supplement: Supplementary file 1 [file ijms-23-11500-s001.zip › ijms-1919716-supplementary.pdf]

SUPPLEMENTARY MATERIAL

Table S1. Statistics related to Figure 2. Top value is the standard error, SE; bottom is the coefficient of determination, $\mathbf{R^2}$, for the curve fit from which the inflection point was calculated to derive the K_D. No statistics could be calculated for experiments that did not yield a binding curve and these are left blank.

SARS-CoV-2	Serum Albumin	Cardio-lipin	β2GPI	Platelet Factor 4	Factor II	Factor VIII	Factor IX	vWF	PDE II	Coll 1
S1				0.015<br>0.9540				0.016<br>0.9893	0.032<br>0.9951	0.047<br>0.9968
S2										
RBD										
Envelope										0.038<br>0.9977
Matrix				0.009<br>0.9940						0.035<br>0.9965
Nucleoprotein					0.039<br>0.9919		0.047<br>0.9893			0.043<br>0.9964
Other Viruses										
Adenovirus			0.059<br>0.9829	0.010<br>0.9820				0.012<br>0.9933	0.031<br>0.9614	
Influenza A										
Coxsackie B										
HSV1						0.013<br>0.9864			0.010<br>0.9820	0.067<br>0.7232

Table S2. Statistics related to Figure 6. Top value is the standard error, SE; bottom is the coefficient of determination, $\mathbf{R^2}$, for the curve fit from which the inflection point was calculated to derive the K_D. No statistics could be calculated for experiments that did not yield a binding curve and these are left blank.

Bacteria	Serum Albumin	Cardio-lipin	β2GPI	Platelet Factor 4	Factor II	Factor VIII	Factor IX	vWF	PDE II	Coll 1
Staphylococcus		0.038<br>0.9769	0.083<br>0.9324	0.008<br>0.9974					0.011<br>0.9944	
GAS goat		0.028<br>0.9529		0.009<br>0.9940	0.021<br>0.8405	0.053<br>0.9584		0.007<br>0.9965	0.012<br>0.9850	
GAS rabbit						0.285<br>0.7923				
Klebsiella		0.130<br>0.9749	0.046<br>0.9763						0.014<br>0.9368	
E. coli		0.019<br>0.9837	0.030<br>0.9479	0.003<br>0.9985					0.009<br>0.9915	
Clostridium				0.023<br>0.6745	0.01<br>0.9915					
M. tuberculosis				0.004<br>0.9929	0.032<br>0.9911					

Table S3. Statistics related to Figure 11. Top value is the standard error, SE; bottom is the coefficient of determination, R^2, for the curve fit from which the inflection point was calculated to derive the K_D. No statistics could be calculated for experiments that did not yield a binding curve and these are left blank.

SARS-CoV-2	Strep Gt	GAS RabHRP	Staph Gt HRP	Kleb Gt HRP	E. coli Gt	Clost HRP Rabbit	M. tb GP
S1	0.010<br>0.9793	0.01<br>0.5883	0.002<br>0.9392				
S2	0.008<br>0.9925	0.014<br>0.8170	0.009<br>0.9944	0.04<br>0.8917			
RBD	0.015<br>0.9435	0.005<br>0.8515					
Envelope							
Matrix							
Nucleocapsid							
Adenovirus Gt							
Influenza A							
CVB blend	0.011<br>0.9889		0.002<br>0.9392		0.016<br>0.9746	0.006<br>0.9748	
HSV1			0.017<br>0.9208				
